# Severe cellulitis from methicillin-resistant *Staphylococcus aureus* (MRSA) in a couple of preterm twins: a case report

**DOI:** 10.1186/s13052-024-01659-0

**Published:** 2024-04-19

**Authors:** Noemi Zampatti, Irene Bonato, Andrea Calandrino, Carolina Saffioti, Alessandro Parodi, Giorgia Brigati, Diego Minghetti, Luca Antonio Ramenghi

**Affiliations:** 1 Department of Neuroscience, Rehabilitation, Ophtalmology, Genetics, Maternal and Child Health, University of Genoa, Genoa, 16132 Italy; 2 Department of Maternal and Neonatal Health, Neonatal Intensive Care Unit, IRCCS Istituto Giannina Gaslini, Genoa, 16147 Italy; 3 Department of Pediatrics, Infectious Disease Unit, IRCCS Istituto Giannina Gaslini, Genoa, 16147 Italy

**Keywords:** MRSA, Skin infection, Cellulitis, Abscess, Preterm infants, Case report

## Abstract

**Background:**

Preterms are at risk of systemic infections as the barrier function of their immature skin is insufficient. The long period of hospitalization and the huge number of invasive procedures represent a risk factor for complications. Among the nosocomial infections of the skin, methicillin-resistant *Staphylococcus aureus* (MRSA) is associated with significant morbidity and mortality.

We report a clinical case of cellulitis and abscess in two preterm twins caused by MRSA in a tertiary level Neonatal Intensive Care Unit (NICU).

**Case presentation:**

Two preterm female babies developed cellulitis from MRSA within the first month of extrauterine life. The first one (BW 990 g) showed signs of clinical instability 4 days before the detection of a hyperaemic and painful mass on the thorax. The second one (BW 1240 g) showed signs of clinical instability contextually to the detection of an erythematous, oedematous and painful area in the right submandibular space. In both cases the diagnosis of cellulitis was confirmed by ultrasound. A broad spectrum, multidrug antimicrobial therapy was administered till complete resolution.

**Conclusions:**

Due to the characteristic antibiotic resistance of MRSA and the potential complications of those infections in such delicate patients, basic prevention measures still represent the key to avoid the spreading of neonatal MRSA infections in NICUs, which include hand hygiene and strict precautions, as well as screening of patients for MRSA on admission and during hospital stay, routine prophylactic topical antibiotic of patients, enhanced environmental cleaning, cohorting and isolation of positive patients, barrier precautions, avoidance of ward crowding, and, in some units, surveillance, education and decolonization of healthcare workers and visiting parents.

## Background

Neonates born at < 28 weeks of gestational age and < 1000 g lack enough quantity of vernix caseosa, which has several overlapping functions, like prevention of water loss, regulation of body temperature, maintenance of the innate immunity and an important antimicrobial role. Histologically, the epidermal development completes at the reaching of the 34th week of gestation, for this reason the skin barrier function of preterms is significantly compromised, making them susceptible to thermal, mechanical, and chemical injury as well as to water loss, with elevated risk of severe dehydration, electrolyte imbalance and infections [1, 2].

Thus, those risks are exponentially augmented during the first weeks of life as these infants are frequently exposed to environmental stressors, injuries, and placement of vascular accesses and medical devices [3].

The injured epidermis is a portal of entry for infectious agents and the breakdown of the barrier function of the skin is a risk factor for nosocomial sepsis and death [4–6].

Staphylococcus aureus is a Gram-positive, coagulase-positive pathogen that frequently colonizes skin, skin glands, and mucous membranes of healthy individuals [7, 8]. Methicillin-resistant *Staphylococcus aureus* (MRSA) is characterized by an altered penicillin-binding protein (PBP) with decreased affinity for most semisynthetic penicillins [9]. It is associated with significant morbidity and mortality, especially in extremely preterm infants [10]. Although new antibiotics have been approved, vancomycin remains the agent of choice to treat MRSA infections in children, despite its decreasing susceptibility and toxicity [11].

MRSA infections at both Neonatal Intensive Care Units and community hospitals has become a major problem worldwide [12]. MRSA colonisation has become a constant challenge in healthcare institutions all over the world. Colonisation may easily progress to infection, ranging from only skin infections to sepsis, especially in newborns who need intensive care support [13].

We report a clinical case of cellulitis and abscess in a couple of preterm twins caused by MRSA in a tertiary level Neonatal Intensive Care Unit.

## Case presentation

These female babies were born at the 28th gestational week by caesarean section due to a Twin to Twin Transfusion Syndrome (TTTS) developed in a twin monochorionic biamniotic pregnancy. Empiric antibiotic therapy with gentamicin 5 mg/kg every 48 h and ampicillin 100 mg/kg twice daily was started at birth and discontinued after 5 days, when the blood culture performed at birth resulted negative as well as C-reactive Protein (CRP). Antifungal prophylaxis with fluconazole 3 mg/kg trice weekly was administrated since the first day of life (DOL 1).

### Donor twin (birth weight 990 g)

**Fig. 1:**
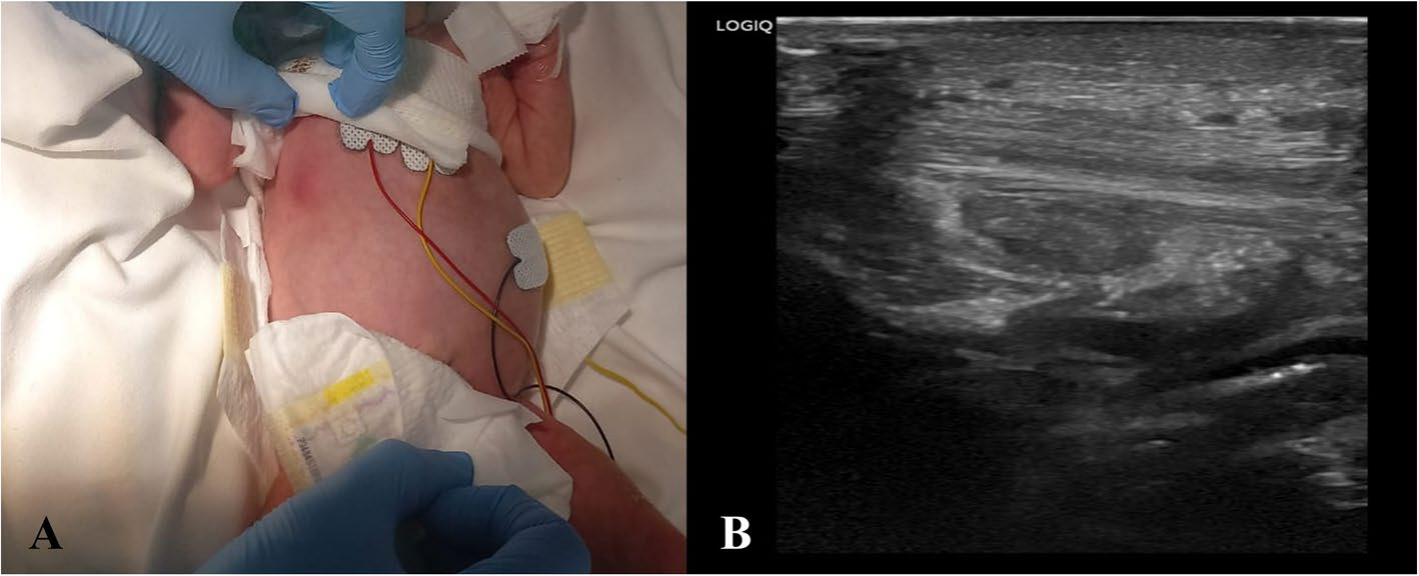
Case #1 clinical observation of a hyperaemic, painful, swelling of taut and mobile consistency mass appeared on the thorax of the baby, in correspondence to the twelfth right rib (**A**). Ultrasound scan of the mass which shows an oval image with a major axis of about 1.3 cm, with a hypoechoic eco-structure, finely corpuscular (**B**)

At DOL 14, a positive screening nasal swab for MRSA was found. A topical therapy with nasal mupirocin trice daily for seven days was administrated. To prevent the spread of MRSA infection, the following day the patient was isolated and followed by a dedicated nurse. In addition, increased cleanliness of the environment, barrier precautions were put in place and crowding of the ward was avoided. Screening swabs were performed every four days in the other inpatients. A carrier could not be identified. At DOL 21, the patient developed respiratory distress, bradycardia, and desaturations. Diagnostic workup for neonatal sepsis was performed (blood count, CRP, blood culture and lumbar puncture with chemical physical and cultural analysis of liquor). A moderate leucocytosis and CRP elevation was found. A broad-spectrum empirical antibiotic therapy with vancomycin 15 mg/kg every 12 h and meropenem 40 mg/kg trice daily was started. In our center, based on local epidemiological data, the empirical therapy of late onset sepsis with suspected central nervous system involvement, consists of the combination of vancomycin and meropenem at meningeal dosage. Once the involvement of the nervous system is excluded, the therapy is shifted to vancomycin and piperacillin/tazobactam. At DOL 25 a hyperaemic, painful, swelling of taut and mobile consistency mass, suspected to be a collecting abscess, appeared on the thorax of the baby at the level of the twelfth right rib (Fig. 1, A). Ultrasound scans showed an oval image with a major axis of about 1.3 cm, with a hypoechoic eco-structure, finely corpuscular, without vascular signal, but with peripheral inflammatory vascularization (Fig. 1, B), compatible with an abscess lesion. The antibiotic therapy with vancomycin was then continued (while meropenem administration was discontinued after 7 days, basing on cultural results) and topical sterile medications were administered. After thirteen days of treatment (DOL 33) we assisted to complete clinical improvement and current therapy was discontinued. Since DOL 29 all screening nasal swabs were negative. Also blood and liquor cultures, collected at onset of signs of infection, resulted negative.

### Receiving twin (birth weight 1240 g)

**Fig. 2:**
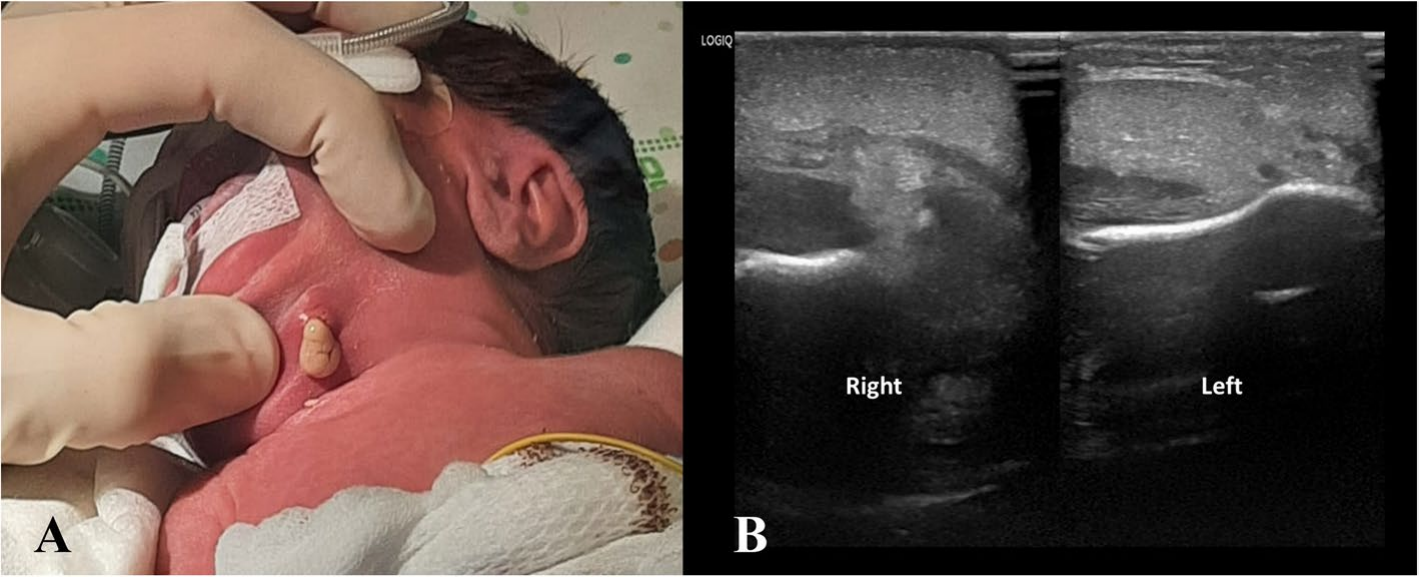
Case #2 drainage of abundant purulent material from the lesion sited in the right submandibular area (**A**). Ultrasound scan with the evidence of tissue inflammation extended up to the subfascial latero-cervical area in the right cervical side, compared to the normal left one (**B**)

At DOL 18, a positive screening nasal swab for MRSA was found. A topical therapy with nasal mupirocin trice daily for seven days was administrated. Again, the same preventive measures were put in place to prevent the spread of MRSA infection. Also in this case a carrier could not be identified. At DOL 20, the patient developed important signs of clinical instability. Diagnostic workup for neonatal sepsis was performed (blood count, CRP, blood culture and lumbar puncture with chemical physical and cultural analysis of liquor). It revealed a marked leucocytosis with important CRP elevation. A broad-spectrum empirical antibiotic therapy with vancomycin 15 mg/kg every 12 h and meropenem 40 mg/kg trice daily was started. On the same day the right submandibular area appeared erythematous, oedematous, and painful (Fig. 2, A). The clinical hypothesis of cellulitis was confirmed by ultrasound, with the evidence of tissue inflammation extended up to the subfascial latero-cervical area (Fig. 2, B). The following days, despite several dosage adjustments of vancomycin on the basis of therapeutic drug monitoring (TDM), due to the impossibility of maintaining adequate levels in order to obtain an AUC/MIC (area under the curve/minimum inhibitory concentration) of 400–600 mg*h/l, a substitutive therapy with teicoplanin 16 mg/kg in the first day, then followed by 8 mg/kg per day was started. At DOL 27, while boric acid packs were in place, the abscess spontaneously drained abundant purulent material (Fig. 2, A), whose microbiological examination revealed MRSA positivity. Also in this case, blood and liquor cultures collected at onset of signs of infection resulted negative. Brain MRI and abdominal ultrasound scan, performed to rule out other abscessual localizations, were negative. After fifteen days of treatment (DOL 35), for persistent drainage of purulent material from the lesion, we decided to continue antibiotic therapy for a further week. Due to the good general clinical conditions, the negativization of CRP and the difficulty in maintaining a stable venous access, we decided to continue an oral therapy with trimetroprim/sulfamethoxazole 7 mg/kg trice daily and rifampicin 10 mg/kg twice daily. Both antibiotics, effective on MRSA on the basis of the antibiogram, were chosen for excellent oral absorption and tissue penetration. All nasal swabs performed two times a week were positive for MRSA colonization. Since DOL 39 all screening nasal swabs were negative.

## Discussion and conclusions

Few cases of cellulitis caused by methicillin resistant Staphylococcus aureus (MRSA) infection in preterm infants are described in literature [14–18]. We report an unusual case of cellulitis caused by MRSA in a couple of preterm twins admitted to the Neonatal Intensive Care Units (NICU).

Due to the importance of a proper microbiological control within the NICU, many clinical and epidemiological reports on dermatological and soft tissue infections already exist for different pathogens, like group B Streptococcus (*Streptococcus agalactiae*) [19, 20] and methicillin-resistant Staphylococcus epidermidis [21]. We recognize the importance of drawing clinicians’ attention to the possible fatal complications of these infections in such complex and delicate patients, it is important to early identify colonised newborns through routine screening nasal or extra-nasal swabs, and to start a prompt topical antibiotic treatment with nasal mupirocin in case of positivity [10]. These patients must be clinically monitored, and a broad spectrum systemic antibiotic therapy must be started as soon as the appearance of signs or symptoms indicative of infection [5].

Preterms and low birth weight newborns are more at risk of complications and death from MRSA skin infection. The prognosis of cellulitis is favourable if it is promptly diagnosed and treated. Otherwise, serious complications may occur, such as subcutaneous abscess, necrotizing fasciitis, osteomyelitis, bacteraemia, septic arthritis, thrombophlebitis, and involvement of eyes, heart valves and central nervous system. Moreover, necrotizing fasciitis usually has a fatal course in 59% of patients [5].

We acknowledge in our second patient the difficulty to reach an adequate vancomycin plasma concentration by administering the internationally recommended drug dosage, it is well known that premature and low birth weight infants may present a wide variability in terms of pharmacokinetical parameters [22, 23]. A different total volume distribution, together with an augmented renal clarence, require individualized dosing regimens to obtain an adequate area under the serum concentration time curve over 24 h/minimum inhibitory concentration ratio in these babies, making more frequent the possibility to change dosage or even molecule [24].

Due to the characteristic antibiotic resistance of MRSA worldwide [25] and uncertain ties in the efficacy and safety of decolonization strategies in neonates, basic prevention measures still represent the key to avoid the spreading of neonatal MRSA infections in NICUs. Hand hygiene and screening for MRSA on admission (nares and umbilicus are considered the most common sites for MRSA colonisation [26]), together with routine prophylactic topical antibiotic therapy in the same sites, have been shown to be the most successful infection control policies to reduce transmission of MRSA [27–29]. Other recommended measures included cohorting and isolation of positive patients, cohorting of nurses for care of MRSA-positive patients, periodic screening of patients in the neonatal unit (weekly to monthly depending on local MRSA transmission rates), enhanced environmental cleaning, barrier precautions and avoidance of ward crowding [30, 31]. In our center, all these measures described in literature were put in place to contain the spread of MRSA infection.

Finally, another strategy suggested in literature to prevent the spreading of neonatal MRSA infection, but not considered in our case because not available for our department, is the use of molecular epidemiological tools to investigate an outbreak [32]. Polymerase chain reaction (PCR)-based screening methods give quicker results and have been shown to lead to a reduction in the rate of MRSA transmission [33]. This represents a promising preventive strategy to be used where available.

## Data Availability

All datasets generated and analyzed during the current study are not publicly available but are available from the corresponding author on reasonable request. Raw data were generated at IRCCS Istituto Giannina Gaslini. Individual patient data will be made available on request in agreement with data privacy statement signed by the parents.

## Abbreviations

BW: Birth Weight
CRP: C-Reactive Protein
MRSA: Staphylococcus Aureus Meticillino-Resistant
TTTS: Twin to Twin Transfusion Syndrome
DOL: Day Of Life
NICU: Neonatal Intensive Care Unit
MRI: Magnetic Resonance Imaging
TDM: Therapeutic Drug Monitoring
AUC: Area Under the Curve
MIC: Minimum Inhibitory Concentration
